# CASE REPORT: Serial Cases of False-Positive Flow-Cytometry T Cell Crossmatch Associated With Anti–Blood Type Antibodies in Patients Undergoing ABO-Incompatible Kidney Transplantation

**DOI:** 10.3389/fimmu.2022.862652

**Published:** 2022-03-10

**Authors:** Ayaka Hayashi, Izumi Yamamoto, Mayuko Kawabe, Akimitsu Kobayashi, Makoto Ito, Kiyohiko Hotta, Nobuo Shinohara, Tetsunori Tasaki, Takashi Yokoo, Daiki Iwami

**Affiliations:** ^1^Division of Nephrology and Hypertension, Department of Internal Medicine, The Jikei University School of Medicine, Tokyo, Japan; ^2^Division of Laboratory and Transfusion Medicine, Hokkaido University Hospital, Sapporo, Japan; ^3^Department of Urology, Hokkaido University Hospital, Sapporo, Japan; ^4^Department of Transfusion Medicine and Cell Therapy, The Jikei University Hospital, Tokyo, Japan; ^5^Division of Renal Surgery and Transplantation, Jichi Medical University, Shimotsuke, Japan

**Keywords:** kidney transplantation, blood type antigen, crossmatch flow cytometry, HLA crossmatch, Coombs test

## Abstract

**Background:**

A positive flow-cytometry T cell crossmatch (FTXM) has important prognostic implications, even when the complement-dependent cytotoxicity crossmatch is negative. Recent studies have shown that ABO incompatibility is associated with positive FTXM, but the underlying mechanism remains poorly understood.

**Cases:**

In five ABO blood type O recipients of kidneys from wives with type B, FTXM was positive but complement-dependent cytotoxicity crossmatch was negative. Application of a solid-phase technique (LABScreen) revealed no case with antibodies to donor-specific human leukocyte antigen. After removal of type B antibodies from patient sera, FTXM was negative for all five patients. In one tested case, the eluate prepared from the donor’s T lymphocyte agglutinated only type B red blood cells, implying the existence of blood type B substances on donor T lymphocytes.

**Discussion:**

False-positive FTXM reflects blood type B substrates bound to T lymphocytes. Repeat FTXM after incubation with donor-type red blood cells (to adsorb anti-ABO antibodies) was negative. This phenomenon explains the discrepancy between FTXM and solid-phase bead assays. Demonstration of type B substances on donor T lymphocytes is necessary before absolute test validity is confirmed.

**Conclusion:**

False-positive FTXM may be associated with type B antibodies bound to T lymphocytes when a blood type O recipient receives tissue from a type B donor. This phenomenon explains the false-positive FTXM observed in the setting of ABO-incompatible kidney transplantation.

## Background

Given the extreme shortage of transplant organs, performance of immunologically high-risk kidney transplantation in ABO blood type–incompatible and donor-specific antibody (DSA)-positive settings is increasing. Especially in Japan, after overcoming the immunological barrier, ABO blood type–incompatible kidney transplantation today accounts for approximately 40% of all transplantations. However, pre-existing DSA significantly compromises graft outcomes. Risk determination in advance of transplantation *via* histocompatibility testing is essential (1–3). Flow-cytometry T cell crossmatch (FTXM) involves the use of living-donor T lymphocytes to detect anti-donor human leukocyte antigen (HLA) immunoglobulin (Ig) G antibodies, but the results can be affected by non-HLA antigens on T lymphocyte cell surfaces. A recent study demonstrated that ABO incompatibility was associated with FTXM positivity in recipients with ABO blood type O, but the underlying mechanism remains poorly understood (4). Herein, we report on five living-donor kidney-transplant patients with false-positive FTXM results. We show that such false positivity may reflect the presence of incompatible ABO blood type substances on donor T lymphocytes.

## Cases

Between 2009 and 2019, 64 ABO-incompatible kidney transplants were performed at Hokkaido University Hospital, and between 2016 and 2021, twelve were performed at Jikei University Hospital. CDC crossmatch (CDC-XM) and flow-cytometry T cell crossmatch (FTXM) were conducted in all 76 cases. The details of the cases are given in **Table 1**; there were 44 cases of blood group A incompatibility, 27 cases of blood group B incompatibility, and five cases of blood group A and B incompatibility. Among them, the combination of CDC-XM-negative and FTXM-positive was found in five, only in transplants from blood group B donors to blood group O recipients. The five cases described here account for 6.6% (5/76) of the cohort, and 33% (5/15) of blood type O recipients with blood type B donors. All five patients were male, had blood type O, and received kidneys from their wives with blood type B (**Tables 1**, **2**). Cases 1–3 were treated at Hokkaido University Hospital, and cases 4 and 5 were treated at Jikei University Hospital. For cases 1–3, all laboratory procedures were performed in the Division of Laboratory and Transfusion Medicine, Hokkaido University Hospital. For cases 4 and 5, all laboratory procedures were performed in the Department of Transfusion Medicine and Cell Therapy, Jikei University Hospital, except that FTXM was outsourced to Reprocell Inc.

**Table 1:** Distribution of FTXM positive cases in ABOi KTx.

ABO blood type		Cases	
Donor	Recipient	n	FTXM(+)
A	O	23	0
A	B	15	0
AB	B	6	0
B	O	15	5
B	A	9	0
AB	A	3	0
AB	O	5	0
		76	5

FTXM, flow-cytometry T cell crossmatch; ABOi KTx, ABO incompatible Kidney transplantation.

**Table 2:** Demographic and clinical features of FTXM positive cases.

	Case 1	Case 2	Case 3	Case 4	Case 5
**Age**	53	59	69	50	62
**Gender**	Male	Male	Male	Male	Male
**Blood Type**	O	O	O	O	O
**Original Disease**	ADPKD	Obstructive Nephropathy	BNS	ADPKD	BNS
**History of sensitization**	Transfusion	Transfusion	No	No	No
**Co-morbidity**	HCV	IBD	Diabetes Mellitus	Cerebral Infarction	Urolithiasis
**Donor Relationship**	Wife,52 y	Wife,56 y	Wife,56 y	Wife,47 y	Wife,60 y
**Donor Blood Type**	B	B	B	B	B
**HLA mismatch** **(A、B、DRB1 ± DQB1)**	4(8)	2(8)	5(8)	4(6)	6(6)
**Anti-B antibody titer** **Saline/Coombs methods**	×8/×32	×8/×128	×64/×256	×16/×512	×8/×16
**Pre-KTx CDC-XM**	Negative	Negative	Negative	Negative	Negative
**Pre-KTx FTXM** **Case1,2,3:MCS** **(cut-off 20％)** **Case4,5:NC IgG/XM IgG FITC (cut-off 1.4)**	85.7%(positive)	52.8%(positive)	78.7%(positive)	2.2(positive)	1.9(positive)
**LABScreen^®^ single antigen**	DPA1,01:03 MFI=1469	none	none	A80 MFI=2555	none

ADPKD, autosomal dominant polycystic kidney diseases; BNS, Benign Nephrosclerosis; CDC-XM, complement-dependent cytotoxicity crossmatch; ESKD, endo-stage kidney diseases; FTXM, flow-cytometry T cell crossmatch; HCV, hepatitis C virus; HLA, human leukocyte antigen; IBD, inflammatory bowel disease; KTx, kidney transplantation; MCS, median channel shift; NC, negative control, MFI, mean fluorescence intensity.

In cases 1–3, anti–B antibody titers measured using the saline/Coombs methods with dithiothreitol at referral were 1:64/1:256, 1:8/1:64, and 1:8/1:32, respectively. In cases 4 and 5, the titers at referral measured by the saline/Coombs methods without dithiothreitol were 1:16/1:512 and 1:8/1:16, respectively. FTXM results were positive, but lymphocyte cytotoxic assay findings were negative, in all cases. In cases 1–3, the median FTXM mean channel shift (MCS) was 78.7% (range 52.8–85.7%:cut-off 1.4). In cases 4 and 5, the negative control IgG–fluorescein isothiocyanate values were 2.2 and 1.9, respectively (cut-off 1.4). The numbers of mismatched HLAs (including HLA-A, -B, -DRB1, and DQB1) for cases 1–3 were 4, 2, and 5, respectively (HLA-DP was not typed). The numbers of mismatches (including HLA-A, -B, and -DRB1) in cases 4 and 5 were 4 and 6, respectively (HLA-DQ and -DP were not typed). Cases 1 and 5 expressed non-DSAs to HLA-DPA1-01:03 (mean fluorescence intensity [MFI] 1,469) and HLA-A80 (MFI 2,555), respectively; a solid-phase technique (LABScreen) revealed no anti-HLA IgG antibodies in the other cases, implying a lack of DSAs. These results imply that non-HLA antigens on T lymphocytes might be the cause of the FTXM positivity. We hypothesized that a blood type B substance on T lymphocytes rendered FTXM results (falsely) positive.

**Figure 1:**
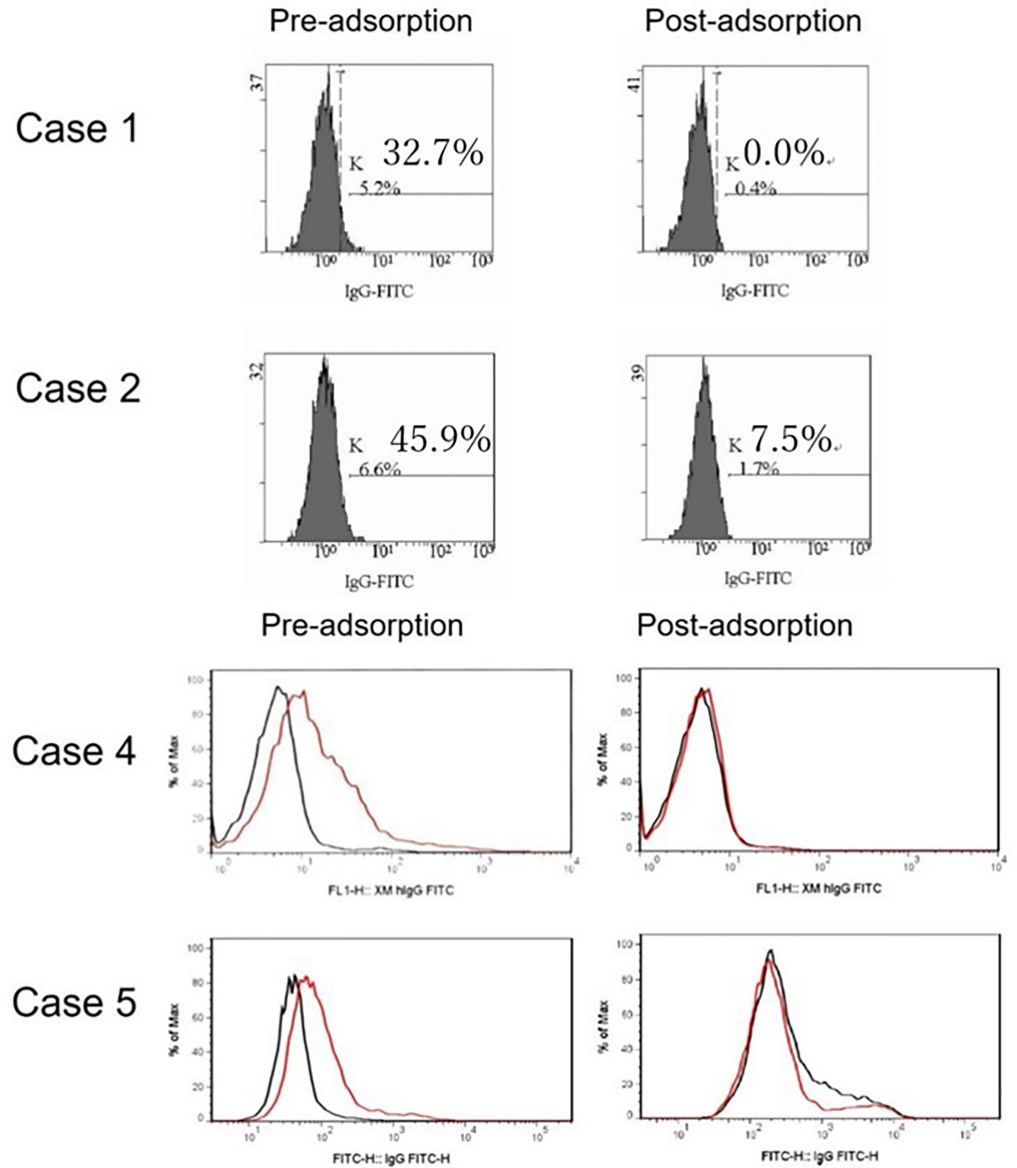
FTXM results for kidney-transplant recipients obtained before and after preincubation with donor red blood cells (RBCs). Recipient sera were incubated with donor type B RBCs to adsorb anti-ABO antibodies prior to repeat FTXM; all results were then negative. In cases 1 and 2, the MCS values changed from 32.7% to 0.0% and 45.9% to 7.5%, respectively. In cases 4 and 5, the negative control IgG-FITC (minute XM IgG-FITC) values fell from 1.9 to 1.0 and 2.2 to 1.1 (cutoff 1.4), respectively.

**Figure 2:**
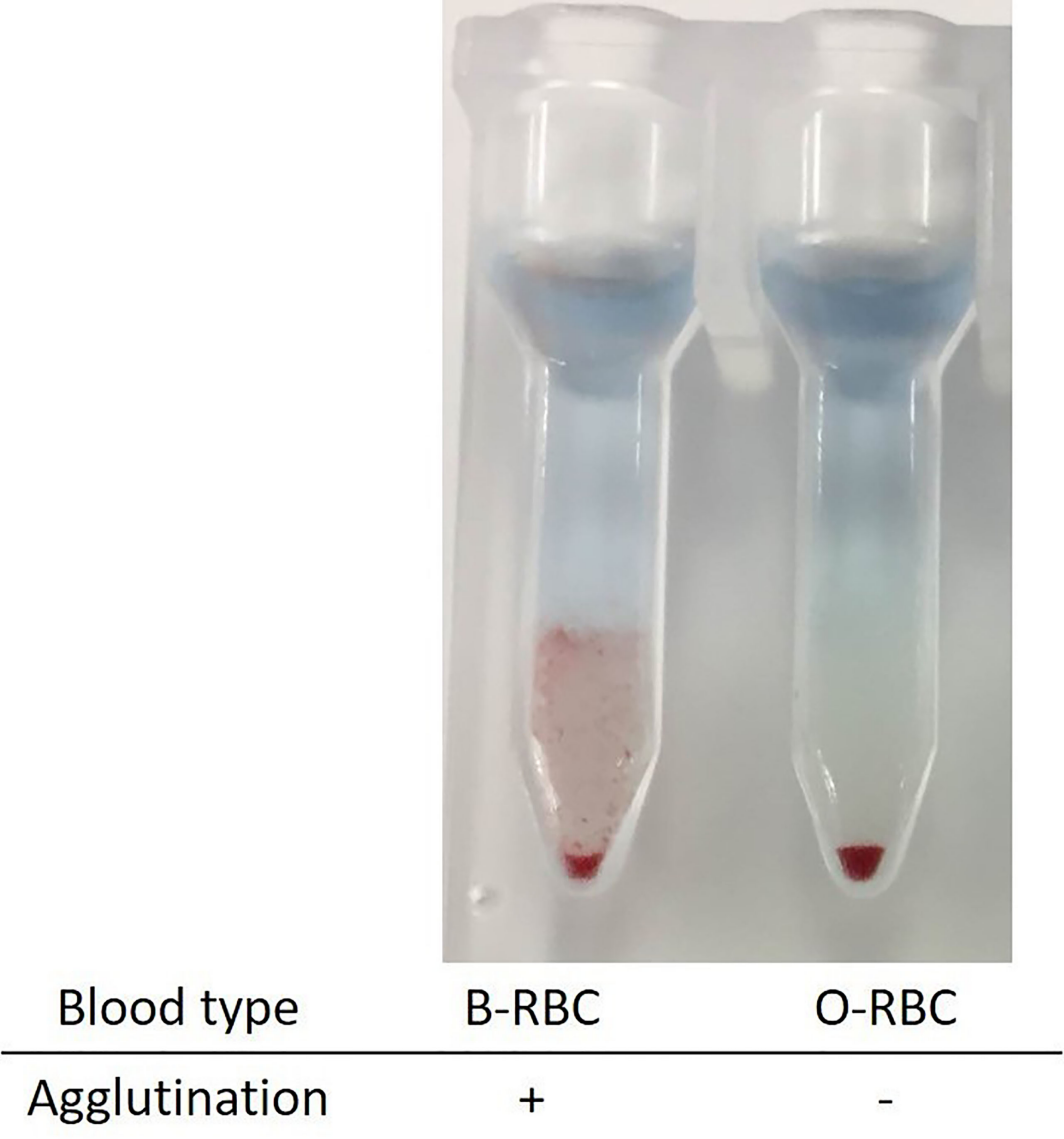
Indirect Coombs test with the eluates from donor T lymphocyte. Donor lymphocytes were incubated with recipient sera, and attached antibodies were extracted into an acidic solution. The eluate was mixed with red blood cells of types B and O. Only the former agglutinated, implying the existence of type B material on the donor T lymphocytes.

To explore this hypothesis, recipient sera were preincubated with type B red blood cells (RBCs) to adsorb anti–blood type antibodies; then, repeat FTXM with donor T lymphocytes was carried out using the sera (Experiment 1). Repeat FTXM results were negative in all patients, implying that a DSA (an anti–blood type IgG antibody) was detected by the original FTXM (**Figure 1**). We then extracted antibodies binding to donor T lymphocytes and performed the indirect Coombs test. For case 3, we incubated donor T lymphocytes with recipient serum and then eluted cell-bound antibodies using an acidic solution (Experiment 2) (5). The eluate was incubated with donor-type or non–donor-type RBCs (the indirect Coombs test). The eluate agglutinated blood type B, but not type O, RBCs (**Figure 2**), indicating that type B IgG antibodies were bound to the donor T lymphocytes. This phenomenon explained the FTXM positivity.

We concluded that all five patients lacked DSAs apart from anti–B IgG antibodies. We performed desensitization therapy followed by ABO-incompatible kidney transplantation. Only case 2 developed acute antibody-mediated rejection (presumably caused by anti-B antibodies) on the day after transplantation; we initiated anti-rejection therapy and the patient is currently under observation with stable kidney function.

### Experiment 1

To remove anti-B antibodies from patient sera, patient sera were mixed with washed blood type B RBCs at a 2:1 (v/v) ratio, incubated at 37°C for 1 h, and centrifuged at 3,000 rpm for 5 min. The supernatants were collected and FTXM was performed. We compared the results with and without anti–B antibody absorption.

### Experiment 2

Donor T lymphocytes were incubated with recipient sera containing anti-B antibodies. The T lymphocytes were collected and the bound antibodies were eluted into an acidic solution, which was mixed with type B and type O RBCs. If anti–B antibodies were present in the eluate, only type B RBCs would agglutinate.

## Discussion

FTXM is more sensitive than complement-dependent cytotoxicity crossmatch (CDCXM); it aids in identification of optimal donors and desensitization prior to kidney transplantation. FTXM positivity has important prognostic implications, even when CDCXM results are negative (1–3). Thus, interpretation of positive FTXM results has become critical. Here, we describe five kidney-transplant recipients of blood type O who received kidneys from their blood type B wives; FTXM results were positive but CDCXM and DSA test results were negative in these cases. After absorption of anti–B antibodies from recipient sera, FTXM results became negative (Experiment 1) and anti-B antibodies were eluted from donor T lymphocytes which had been incubated with recipient’s serum (Experiment 2). Recently, Lindemann et al. analyzed 29 ABO-incompatible and 89 ABO-compatible donor–recipient pairs and found that ABO incompatibility was associated with higher FTXM responses, especially in recipients of blood group O; they suggested that this phenomenon was attributable to donor-specific IgG isohemagglutinin (4). Furthermore, we found that false-positive FTXM results were attributable to type B antibodies bound to T lymphocytes. To our knowledge, this report is the first to suggest that ABO type B antibody (a non-HLA DSA) explains FTXM positivity in solid organ recipients.

In terms of the precise mechanism in play, it is reasonable to suggest that ABO antigens exist on donor T lymphocytes. The relationship between ABO status and the Lewis blood type antigen system is complex. ABO and Lewis antigens are synthesized from a common precursor. ABO antigens are synthesized in and expressed on RBCs (6). In contrast, Lewis antigens are synthesized *via* the action of fucosyltransferase [FUT] 3 in cells other than RBCs, such as in salivary glands; secreted into fluids such as saliva, semen, urine, and plasma *via* the action of FUT2; and then become adsorbed onto the surfaces of various cells, including RBCs (7). Notably, secreted ABH substances are also adsorbed onto T lymphocytes in patients with the secretory phenotype. When such lymphocytes are incubated with plasma from patients with the secretory phenotype containing glycosphingolipid fraction, ABH substances are adsorbed onto them, resulting in CDCXM positivity (8, 9). Notably, patients with the FUT3^-^/FUT2^+^ (i.e., Lewis-negative and secreting) phenotype do not synthesize Lewis antigens; rather, they synthesize higher-than-normal levels of ABH substances, which are secreted into the plasma and adsorbed onto the surfaces of cells, including T lymphocytes (10). Similarly, Mittal et al. reported that a non-HLA serum was cytotoxic to lymphocytes from patients who were of blood group B, Lewis negative, and ABH(O) secretors (11). Thus, for case 5, we examined the Lewis antigens on RBCs and the ABH saliva status, and found that this donor was Lewis positive (Le [a- b+]) and an ABH(O) secretor, which is the most common such phenotype among Japanese subjects (data not shown) (12, 13). These results imply that factors other than Lewis negativity and the secretory phenotype are involved.

Theoretically, this phenomenon may occur in blood group A incompatibility as well. However, in our study, there were 44 cases of blood group A incompatibility, rather more than 32 cases of blood group B incompatibility, yet none of them were FTXM-positive. This suggests that the adhesion of blood group antigens on T lymphocytes may be stronger for blood group B antigens than for blood group A antigens. For a more detailed study, it would be useful to prove the presence of blood group A or B antigens by FTXM using antibodies against Acetyl-D-glucosamine (blood group A antigen) and antibodies against D-galactose (blood group B antigen) as primary antibodies.

Many antibodies to non-HLAs are known, but their immunogenicities remain poorly understood. When FTXM and solid-phase bead-assay results disagree, we recommend repeat FTXM after pre-incubation of recipient serum with donor-type RBCs (to adsorb anti–blood type IgG antibodies).

## Limitations

We report on only five patients in this study. We did not prove the existence of ABO blood type B antigen on donor T lymphocytes. Further work is required.

## Conclusion

We encountered five instances of false-positive FTXM results that became negative after preincubation of sera with donor-type RBCs, implying that type B substance on donor T lymphocytes reacted with anti-B antibodies in recipient sera.

## Data Availability Statement

The original contributions presented in the study are included in the article/supplementary material. Further inquiries can be directed to the corresponding author.

## Ethics Statement

Ethical review and approval was not required for the study on human participants in accordance with the local legislation and institutional requirements. The patients/participants provided their written informed consent to participate in this study.

## Author Contributions

AH and IY worked in the clinics, co-designed the study protocol, and co-drafted the manuscript. MK, AK, KH, NS, and TY collected and analyzed the data. IM designed and performed the experiments. TT (a divisional director) performed the data analysis, made valuable suggestions, and supervised the work. TY (a divisional director) also supervised the work. DI (a divisional director) worked in the clinic, co-designed the study protocol, co-drafted and reviewed the manuscript, and supervised the work. All authors contributed to manuscript preparation and approved the final version.

## Conflict of Interest

The authors declare that the research was conducted in the absence of any commercial or financial relationships that could be construed as a potential conflict of interest.

## Publisher’s Note

All claims expressed in this article are solely those of the authors and do not necessarily represent those of their affiliated organizations, or those of the publisher, the editors and the reviewers. Any product that may be evaluated in this article, or claim that may be made by its manufacturer, is not guaranteed or endorsed by the publisher.
